# Supplementary material for: Integrated Proteomics and Lipidomics Investigation of the Mechanism Underlying the Neuroprotective Effect of N-benzylhexadecanamide
Source: Molecules. 2018 Nov 9;23(11):2929. doi: 10.3390/molecules23112929 (PMC6278518; doi:10.3390/molecules23112929)
Supplement: Supplementary file 1 [file molecules-23-02929-s001.pdf]

Integrated Proteomics and Lipidomics Investigation of the Mechanism Underlying the Neuroprotective Effect of N-benzylhexadecanamide

Yanyan Zhou[1] Hongjie Wang[1] Feifei Guo[1] Nan Si[1] Adelheid Brantner[2] Jian Yang[1] Lingyu Han[1] Xiaolu Wei[1] Haiyu Zhao[1*] & Baolin Bian[1*]

[1]Institute of Chinese Materia Medica, China Academy of Chinese Medical Sciences

[2]Institute of Pharmaceutical Sciences Pharmacognosy, University of Graz

[*]Correspondence authors at: Institute of Chinese Materia Medica, China Academy of Chinese Medical Sciences

Prof. Haiyu Zhao, E-mail address: hyzhao@icmm.ac.cn. Tel: 010-64021008; Prof. Baolin Bian, E-mail address: blbian@icmm.ac.cn. Tel: 010-64021008.

Table S1. The results of precision, repeatability and stability

Analyte	precision (RSD%, n=6)	repeatability (RSD%, n=5)	stability (RSD%, n=6)
aspartic acid	2.46	4.23	6.43
asparagine	1.35	4.21	1.51
serine	1.31	4.51	6.73
taurine	3.10	2.02	6.97
tyrosine	1.41	3.29	3.96
noradrenaline	2.05	4.68	5.32
Homovanillic acid	2.75	5.40	3.84
choline	0.70	5.58	3.74
acetylcholine	0.45	4.06	2.36
butyrylcholine	1.07	5.91	4.63

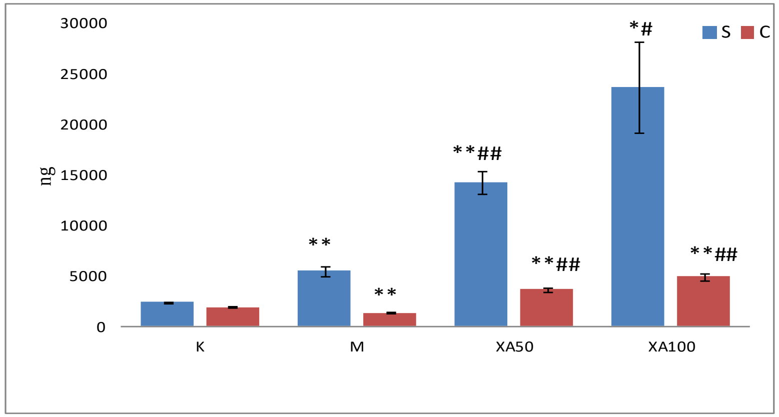

aspartic acid

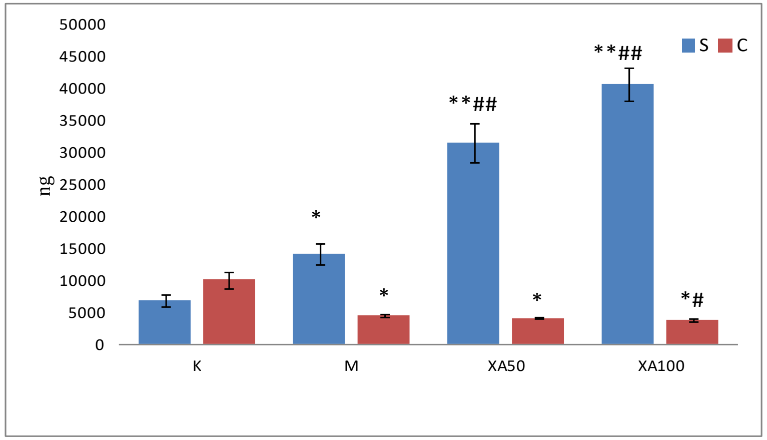

glutamine

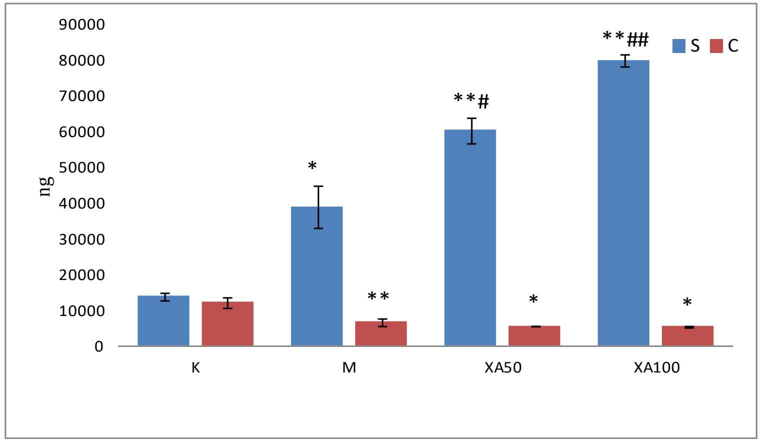

serine

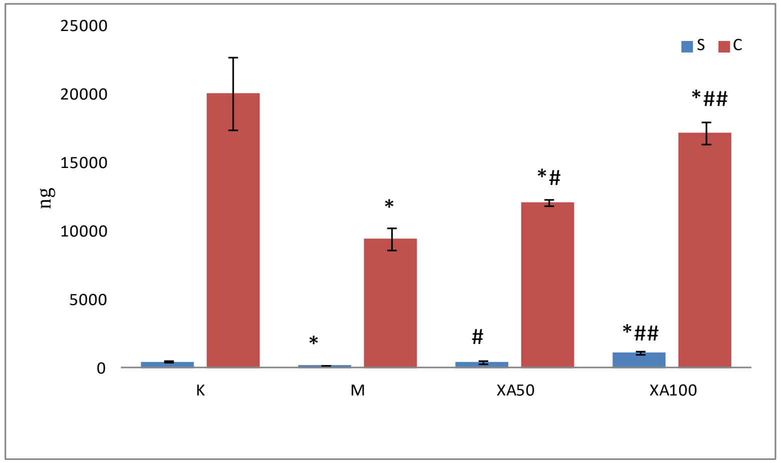

taurine

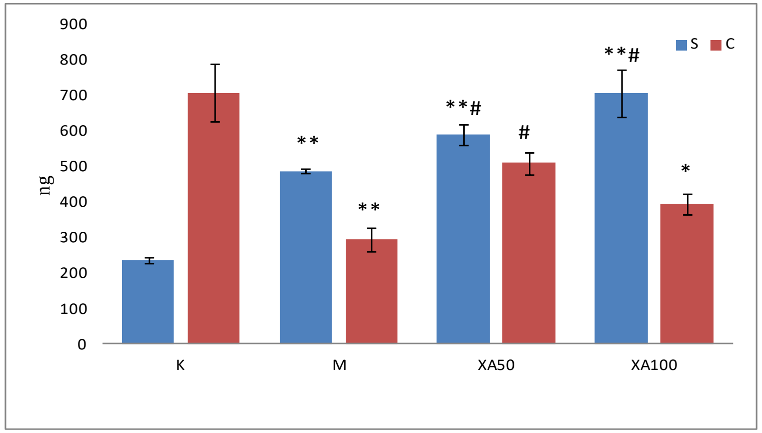

tyrosine

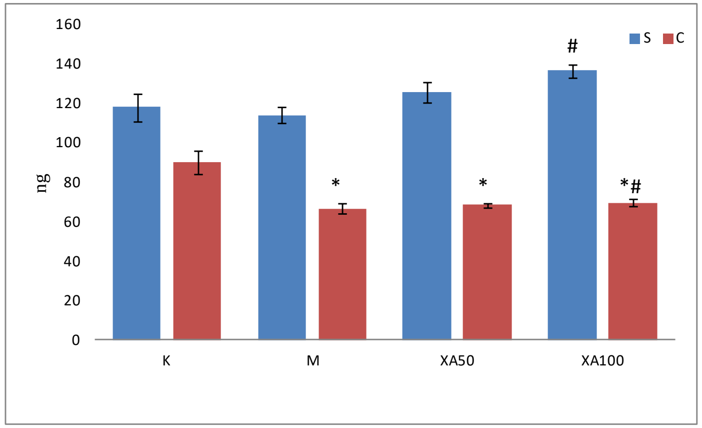

noradrenaline

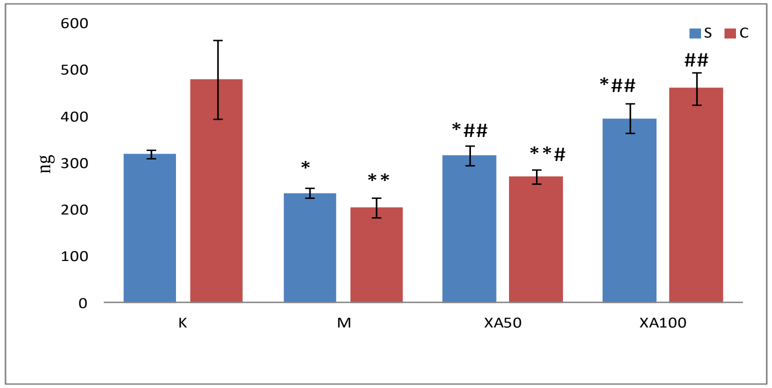

Homovanillic acid

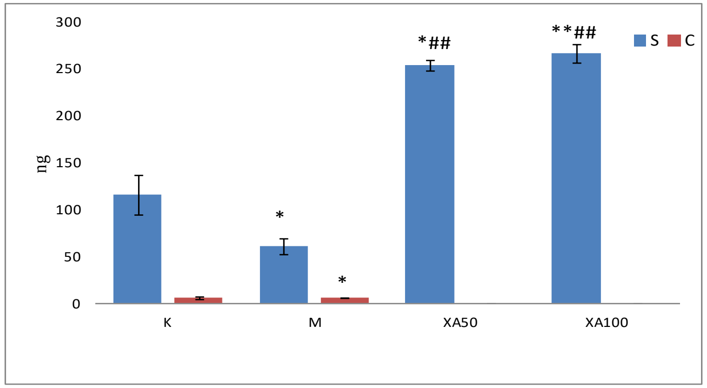

choline

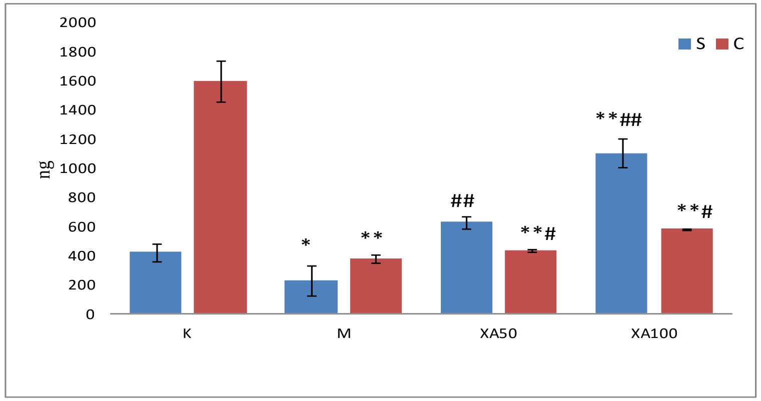

acetylcholine

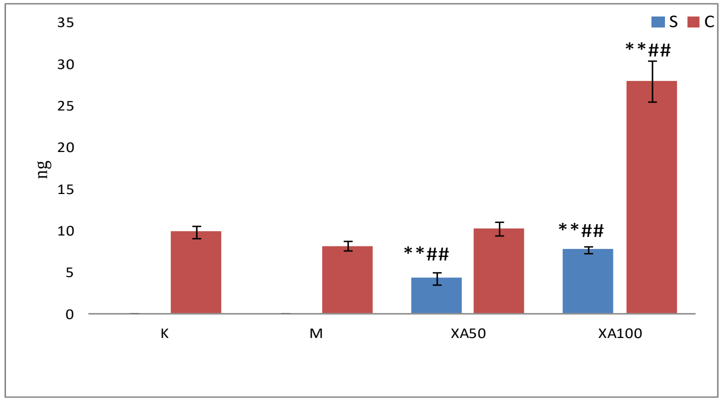

butyrylcholine

Fig. S1 The absolute quantification results of neurotransmitters（(Ax/Ai)×*100, n=3,*P<0.05, **P<0.01: VS controlgroup; #P<0.05, ##P<0.01: VS model group)

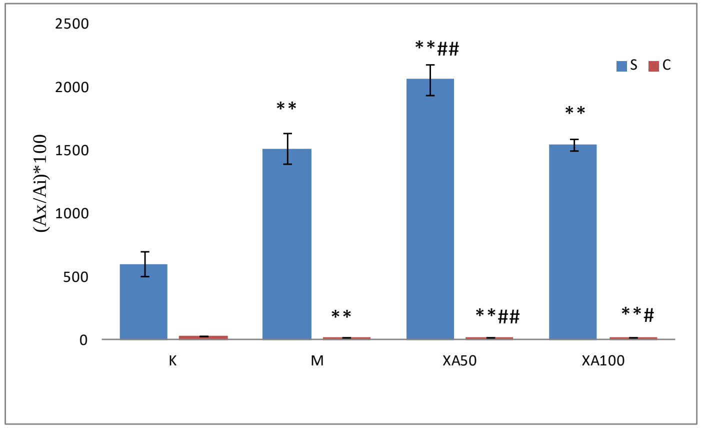

ornithine

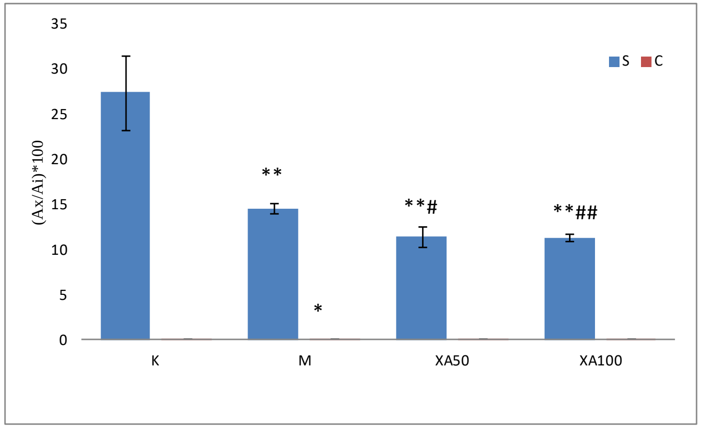

lysine

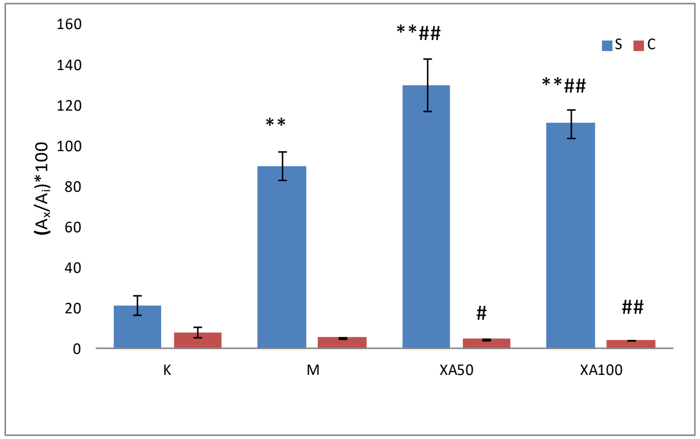

phenylalanine

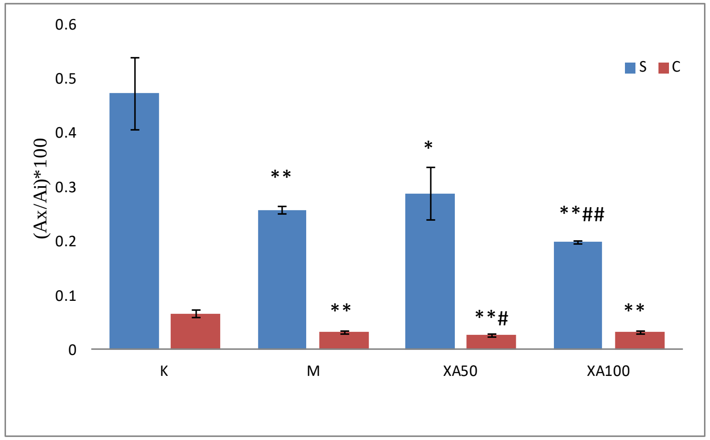

tryptophan

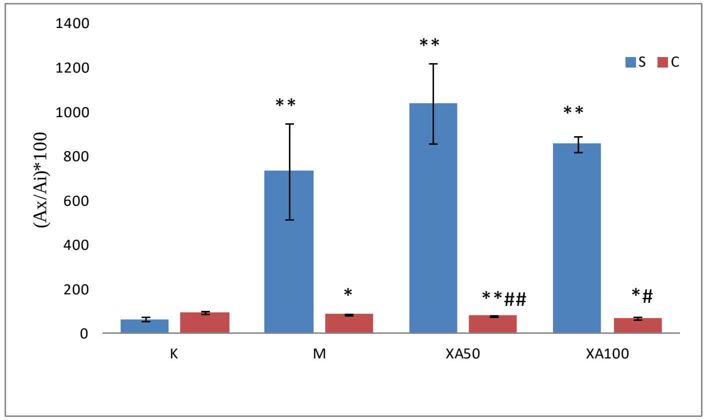

L-leucine

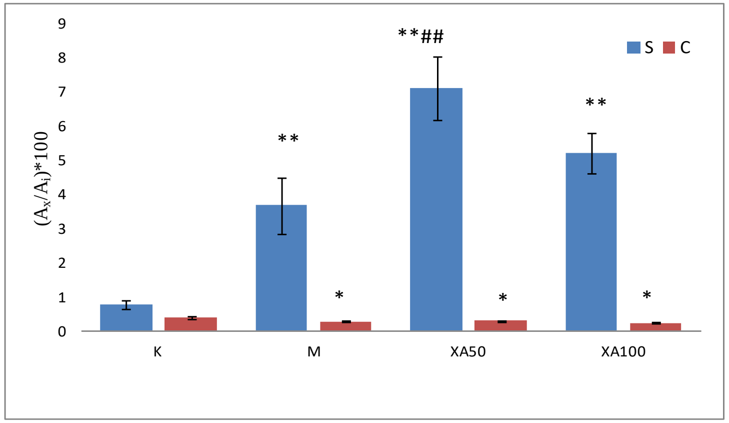

methionine

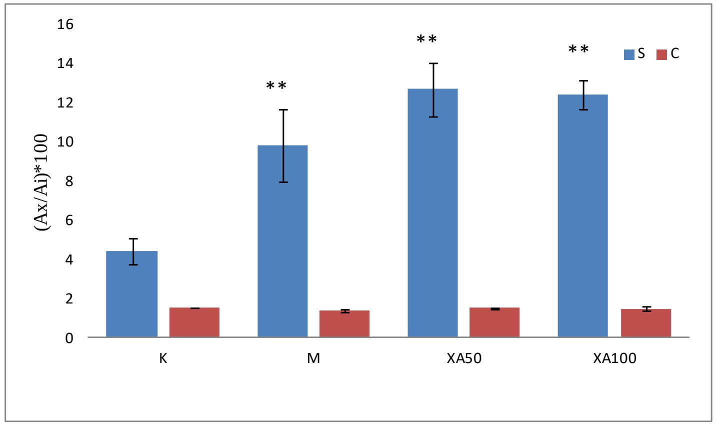

dimethylglycine

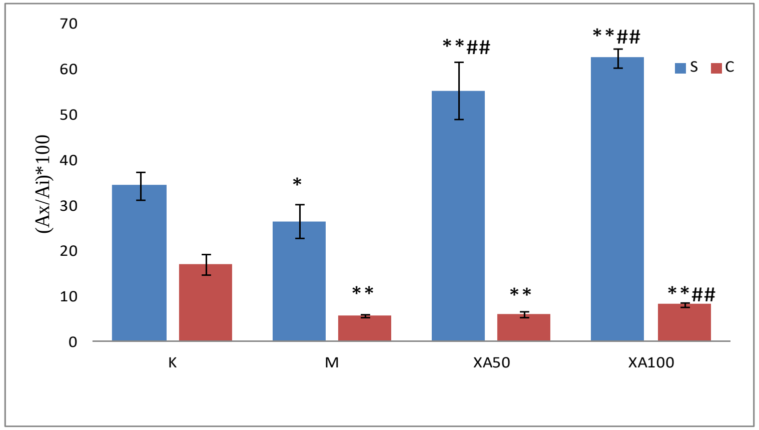

proline

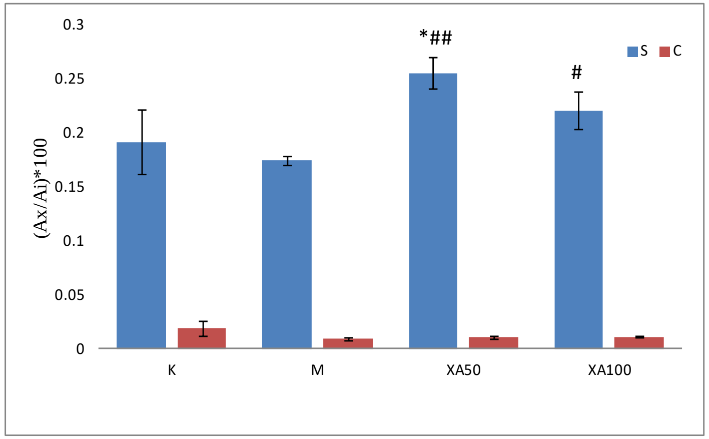

histamine

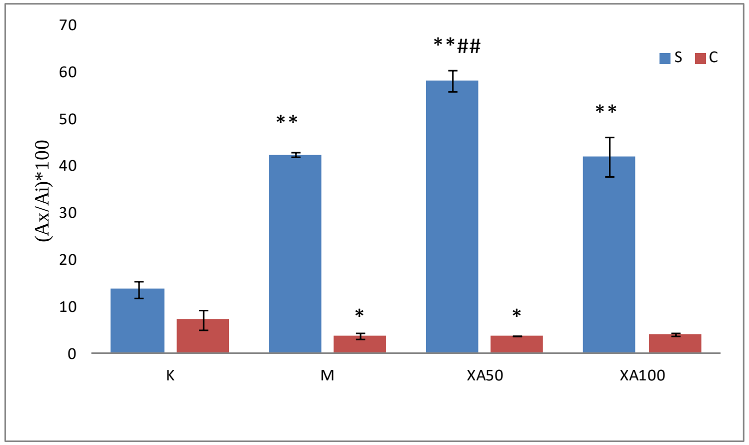

threonine

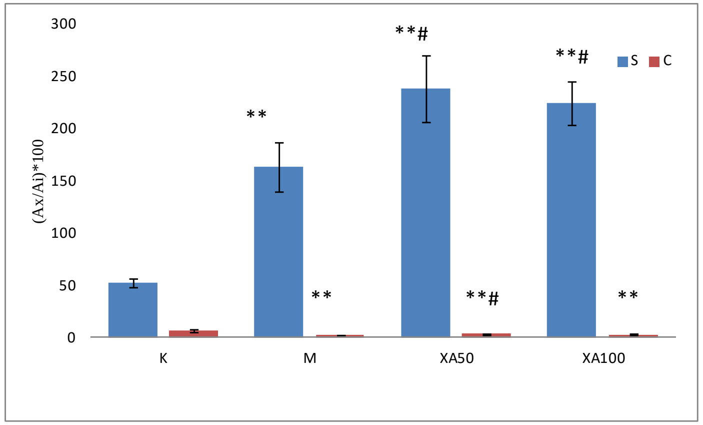

citrulline

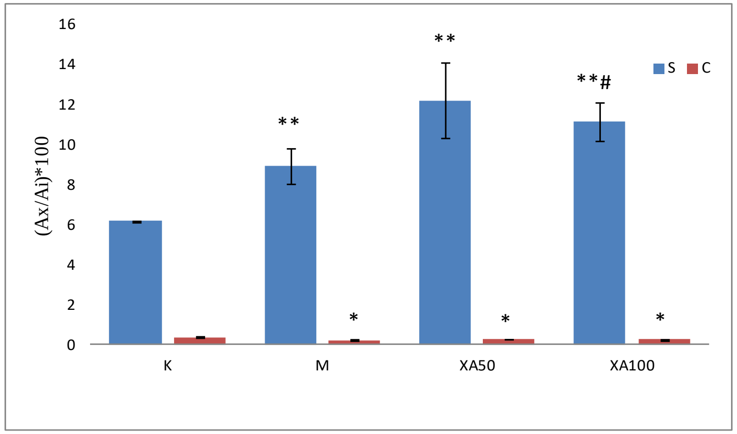

arginine

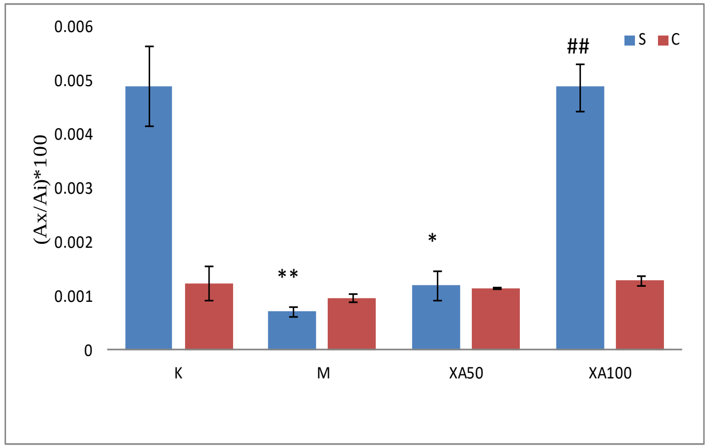

serotonin

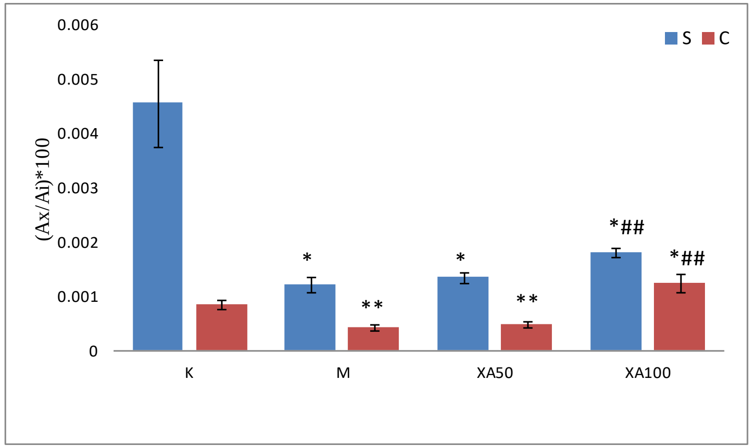

adrenaline

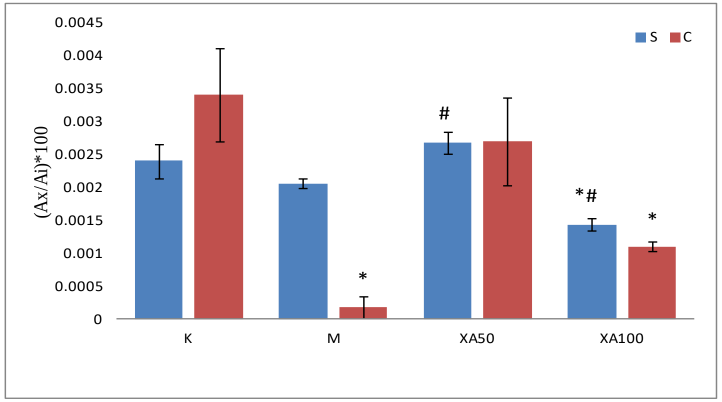

dopamine

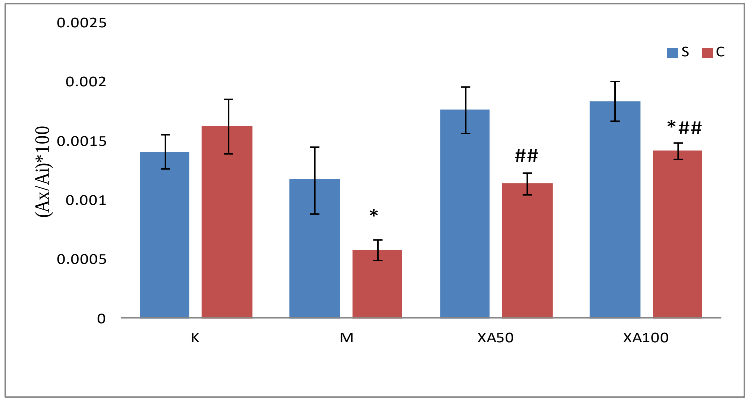

levodopa

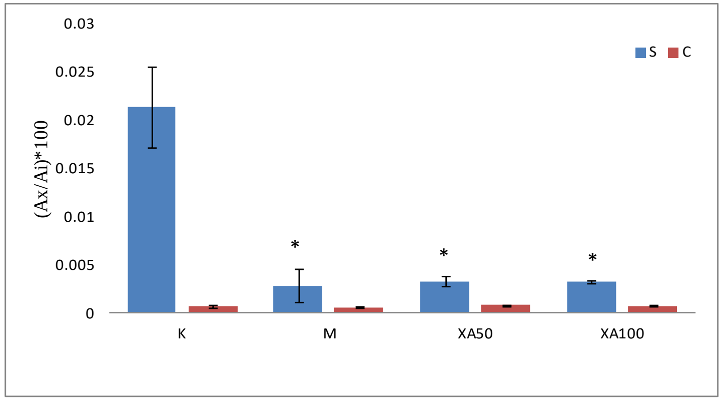

γ-aminobutyric acid

Fig. S2 The relative quantification results of neurotransmitters（(Ax/Ai)*100, n=3, *P<0.05, **P<0.01: VS control group; #P<0.05, ##P<0.01: VS model group

Y-axis: (A_x/A_i)×*100

Table S2. 142 were upregulated and 209 were downregulated after XA treatment

142 proteins upregulated after XA treatment	209 proteins downregulated after XA treatment
SMNDC1	NTPCR
MISMATCH	SLC12A7
DNAJC10	BNIP2
NUP50	GM7244
ASPH	HIRIP3
NUB1	ATPIF1
MISMATCH	LSR
OPA1	TUSC3
NFU1	HNRNPD
POLDIP3	HIF1AN
CNOT1	MAPKAPK2
DHX29	MISMATCH
PKN2	BPHL
PBX3	DIEXF
BAX	PTP4A2
RCOR1	KNTC1
HEATR1	HDAC6
NFIC	TTC9C
FMR1	AMFR
PLCB3	FRYL
YTHDF2	SAT2
COX15	CDC27
D1BWG0212E	1700067P10RIK
AK1	CRTC3
SDF4	RFK
ANAPC4	CSTF2T
ALDOC	SAFB2
CIAPIN1	INSR
PPP4C	MKL2
TOP2B	RUFY3
NDUFB9	ORMDL2

ZC3H14	MTMR6
DHFR	AGFG2
UBQLN2	PDE6A
SCCPDH	LY6G5B
POGZ	OSBPL10
TTC39B	SLC11A2
DDX50	KCTD10
SCARB2	ZFP639
5730455P16RIK	4933407C03RIK
VWF	SETDB1
NPC2	DEF6
ATP5D	PI4KB
EPB4.1L2	AI480653
PRPS2	DDX52
PNMA2	GTF3C4
ATAD3A	MID1IP1
LPCAT1	SUGP2
XRCC6	EVI5L
MFAP1A	MRPL50
CSTB	FGFR1OP
RPS27L	GM4557
CDKN2AIP	SYNGR3
VPS25	BLOC1S1
FAR1	POLE3
DHCR7	PMM1
H2-K1	EPHB3
POLR1C	VPS11
CIRBP	GLE1
PROSC	BIRC5
SRSF9	GTF2H4
CCDC88A	PRL7D1
CPT2	HLTF
EIF4E2	RFX4
PSMD10	HERC2

RAB9	NFIA
KBTBD2	KANK2
YARS2	NUDT9
TRMT6	LLGL1
BCKDHB	ECE1
PPP4R2	ITM2B
WWC2	PRRC2B
MFN2	POLR1D
NDUFAF4	FRMD8
GM15800	RB1CC1
ACYP2	HSDL2
PCM1	ILKAP
TBC1D2B	HSPB6
SLC1A5	SNAPIN
C030046E11RIK	PDLIM4
NDUFA7	SARM1
PCOLCE	RFNG
CYLD	WFS1
IMPA2	9130011E15RIK
SIN3A	HSPA1A
PPP2R5A	MFGE8
SIPA1L1	GNL3L
MACF1	PSTPIP2
TTN	BCL2L1
GOLGA4	MRPS34
DOCK1	BRD3
EED	RABGGTA
LAMP1	ECT2
LUC7L	HMOX1
SMARCD1	BCS1L
FHL3	MOBKL1A
N6AMT2	SSSCA1
UBP1	LSM6
GM14535	4831426I19RIK

LDLRAP1	WDR62
SRSF4	FRK
PPIP5K2	CSTF3
MRPS9	PHLDB1
TBP	MISMATCH
EIF1B	ANKHD1
CCDC92	PARP10
TTC39A	MLYCD
ADO	AFAP1
GCFC1	CNIH4
UTP20	TCF25
PRPF38A	NCDN
FAM135A	SV2B
PCF11	CCDC38
LARP4B	ATG7
RBM4B	SIK3
MED11	LSM12
DDX54	SMAP1
MLF2	TOR1B
OS9	NENF
D19BWG1357E	BYSL
MRPL9	RCL1
FAM53C	ABHD6
VTI1B	TSR1
USP8	SDF2
ECI1	UACA
MTDH	FAM3A
EPN1	SLC39A10
MLST8	EXOC7
MISMATCH	FAM40A
RSL24D1	GALM
MISMATCH	EXOSC3
TRPV5	TPRKB
POLA1	FBXO30

FIGF	UBE2E1
CAMK2D	ZFPL1
CCDC50	ND4
IRF3	CORO1B
GNPDA2	EIF2B4
MAD1L1	FAM125A
MRE11A	FUK
IFT20	RAB34
AATF	PNPLA6
	IQGAP3
	TMED9
	EPN2
	MEA1
	IKBKG
	CPT1A
	NAA20
	UBE2O
	RRM2
	CTIF
	AVL9
	COX4I2
	EXOC1
	SUPT6H
	PNP
	DTD1
	MYO1C
	FAM96B
	VRK1
	CLASP1
	INTS7
	MYCBP
	KIF1A
	DPY30
	MVD

NIP7
PHC2
WHSC2
HEATR5A
NBEA
PLA2G15
ACSL5
APPL1
PARP12
DUT
EDF1
EIF2A
EHD4
PRKAB1
CSNK2A2
CPSF2
GLB1
PCYOX1
RPIA
CLASP2
LAMTOR3
LRRC16A
EXOSC10
MBOAT7
SMARCC2
S100A10
RNGTT
SLC25A32
EHD3
NRCAM
TAP2
COPG2
ATP6V0A1
2310036O22RIK

NBAS
CFDP1
PBK
AGL
PSMD13
GLA
SQRDL
1190002N15RIK

Table ~~S2~~S3. Validation of some differential lipids

No.	Biomarkers	K(peak area)	M(peak area)	XA50(peak area)	XA100(peak area)
1	PC(0:0/18:0)	10079.7±1851.2	52795.7±4144.4	41642.0±3285.9	66487.7±4132.3
2	PC(24:0/0:0)	19082.5±1243.80	11338±322.4	8628.5±311.8	13945.67±627.2
3	PC(P-18:0/0:0)	4154.5±133.6	754.0±75.0	832.5±19.1	1101.0±69.3
4	PC(16:1(9Z)/22:4(7Z,10Z,13Z,16Z))	835346.7±80424.9	1178028.0±79122.5	793231.3±24676.6	1094704±105037.4
5	PC(18:1(9Z)/22:4(7Z,10Z,13Z,16Z))	335539.7±15938.2	405038.0±24576.2	284216.3±977.9	397058.5±37887.5
6	LysoPC(O-18:0)	13281.2±1098.8	1028.3±85.6	1416.0±91.9	1785.0±18.4
7	PE(P-16:0/0:0)	15205.7±1033.1	5318.7±34.6	4000.0±284.3	6520.0±179.6

K: Control group; M: Model group; XA50: 50 μM XA; XA100: 100 μM XA

Table ~~S4~~S4. Validation of 4 differential proteins

No.	Biomarkers	K(ng/mL)	M(ng/mL)	XA50(ng/mL)	XA100(ng/mL)
1	Csnk2a2	0.7096±0.08189	0.8209±0.08527	0.7233±0.05943	0.6632±0.01767
2	Scarb2	266.813±10.887	244.804±19.894	254.403±18.744	289.369±7.504
3	Vti1b	2.741±0.135	2.680±0.0501	2.777±0.0919	2.842±0.0827
4	Bnip2	0.6524±0.0885	1.232±0.0980	0.992±0.0835	0.4984±0.0492

K: Control group; M: Model group; XA50: 50 μM XA; XA100: 100 μM XA; Csnk2a2: Casein kinase II subunit alpha; Scarb2: Lysosome membrane protein 2; Vti1b: Vesicle transport through interaction with t-SNAREs homolog 1B; Bnip2: BCL2/adenovirus E1B 19 kDa protein-interacting protein 2
